# Giant intraosseous (ectopic) meningioma of the temporoparietal calvarium with rapid expansion over 5 years: A case report

**DOI:** 10.1097/MD.0000000000049808

**Published:** 2026-07-17

**Authors:** Mustafa Kaya, Muhammed Ömer Bakaç, Şengül Koçer

**Affiliations:** a Department of Neurosurgery, Sakarya University Faculty of Medicine Education and Research Hospital, Sakarya, Turkey.

**Keywords:** bone invasion, calvarial tumor, ectopic meningioma, extradural meningioma, Intraosseous meningioma, skull neoplasm

## Abstract

**Rationale:**

Intraosseous (ectopic) meningiomas are rare extradural tumors arising within the calvarial bone and account for <1% of all meningiomas. Because their imaging appearance may range from osteolytic to sclerotic or mixed patterns, they can mimic primary bone tumors or metastatic lesions and lead to diagnostic uncertainty.

**Patient concerns:**

A 50-year-old woman presented with a gradually enlarging right temporoparietal scalp mass associated with mild headache for 5 years. The lesion caused marked cosmetic asymmetry without neurological deficit.

**Diagnoses:**

Serial cranial computed tomography demonstrated an expansile right temporoparietal intraosseous calvarial lesion that enlarged from 5.8 × 5.1 × 1.8 cm in 2019 to 17.6 × 14.3 × 4.4 cm in 2024. The mass showed a heterogeneous mixed osteolytic–sclerotic pattern with diploic expansion, cortical thinning, and irregular inner table extension abutting the dura, without a definite intradural component. Histopathological and immunohistochemical evaluation established the diagnosis of ectopic intraosseous meningioma.

**Interventions:**

The patient underwent right temporoparietal craniectomy with complete excision of the involved calvarial bone. The dura appeared uninvolved intraoperatively and was preserved.

**Outcomes:**

Postoperative recovery was uneventful, with no neurological deficit and marked cosmetic improvement. Histopathological analysis showed a benign meningothelial neoplasm with epithelial membrane antigen and progesterone receptor positivity and a low Ki-67 labeling index of approximately 1%. At 1-year follow-up, imaging showed no evidence of recurrence or regrowth.

**Lessons:**

Giant intraosseous meningiomas may demonstrate striking radiologic aggressiveness and rapid calvarial expansion despite benign histology and a low proliferative index. They should be considered in the differential diagnosis of large calvarial masses. Complete surgical excision with preservation of intact dura, when appropriate, may provide favorable early clinical and cosmetic outcomes, although longer follow-up is required to assess long-term tumor control.

## 1. Introduction

Meningiomas are typically intradural tumors derived from arachnoid cap cells. Ectopic or intraosseous meningiomas, however, arise within the skull bone and constitute fewer than 1% of all meningiomas.^[1]^

Their pathophysiology remains debated: hypotheses include sequestration of arachnoid cells in cranial sutures, entrapment during embryogenesis, post-traumatic implantation, or mesenchymal transformation.^[2]^

Radiologically, intraosseous meningiomas may resemble osteoma, fibrous dysplasia, metastatic disease, or malignant bone tumors – leading to delays in diagnosis and management.^[3,4]^ Although most are benign, some lesions exhibit locally aggressive behavior, including cortical destruction and rapid volumetric expansion.^[5]^

We present one of the largest intraosseous meningiomas reported in the literature, demonstrating dramatic growth over 5 years and extensive calvarial involvement, yet with benign histology and excellent surgical outcome.

## 2. Case presentation

A 50-year-old woman presented with a 5-year history of progressively enlarging right temporoparietal swelling and mild headaches. She had no trauma history or systemic illness. Neurological examination was normal.

Written informed consent for publication of this case report and accompanying images was obtained from the patient. According to institutional policy, ethical approval was not required for a single-patient case report.

### 2.1. Imaging findings

**Figure 1.**
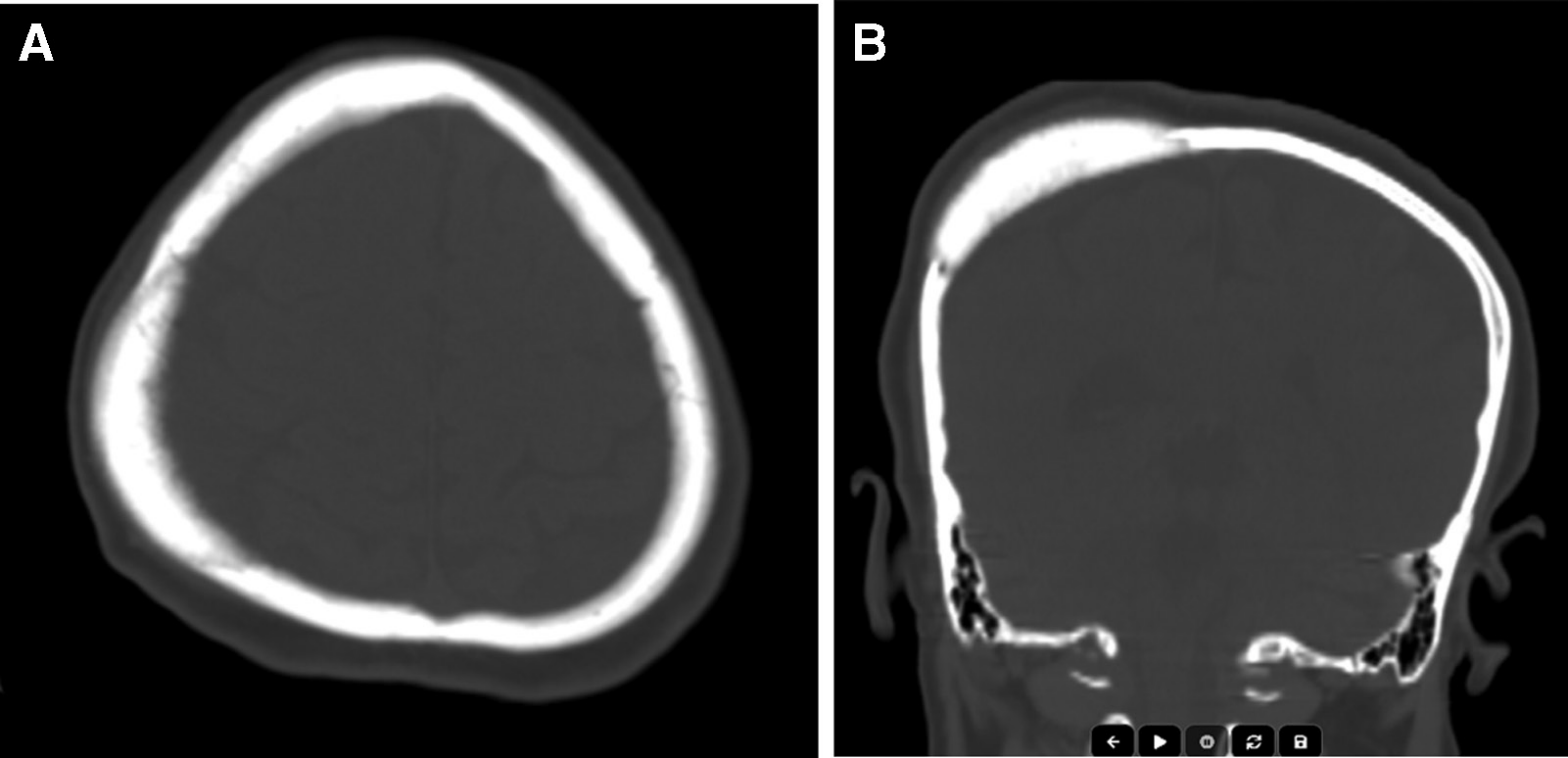
Cranial computed tomography obtained in 2019. (A) Axial bone-window image showing an expansile intraosseous lesion in the right temporoparietal calvarium. (B) Coronal bone-window image demonstrating diploic expansion of the lesion.

A cranial computed tomography (CT) obtained in 2019 demonstrated an expansile intraosseous lesion measuring 5.8 × 5.1 × 1.8 cm within the right temporoparietal calvarium (Fig. 1A, B).

**Figure 2.**
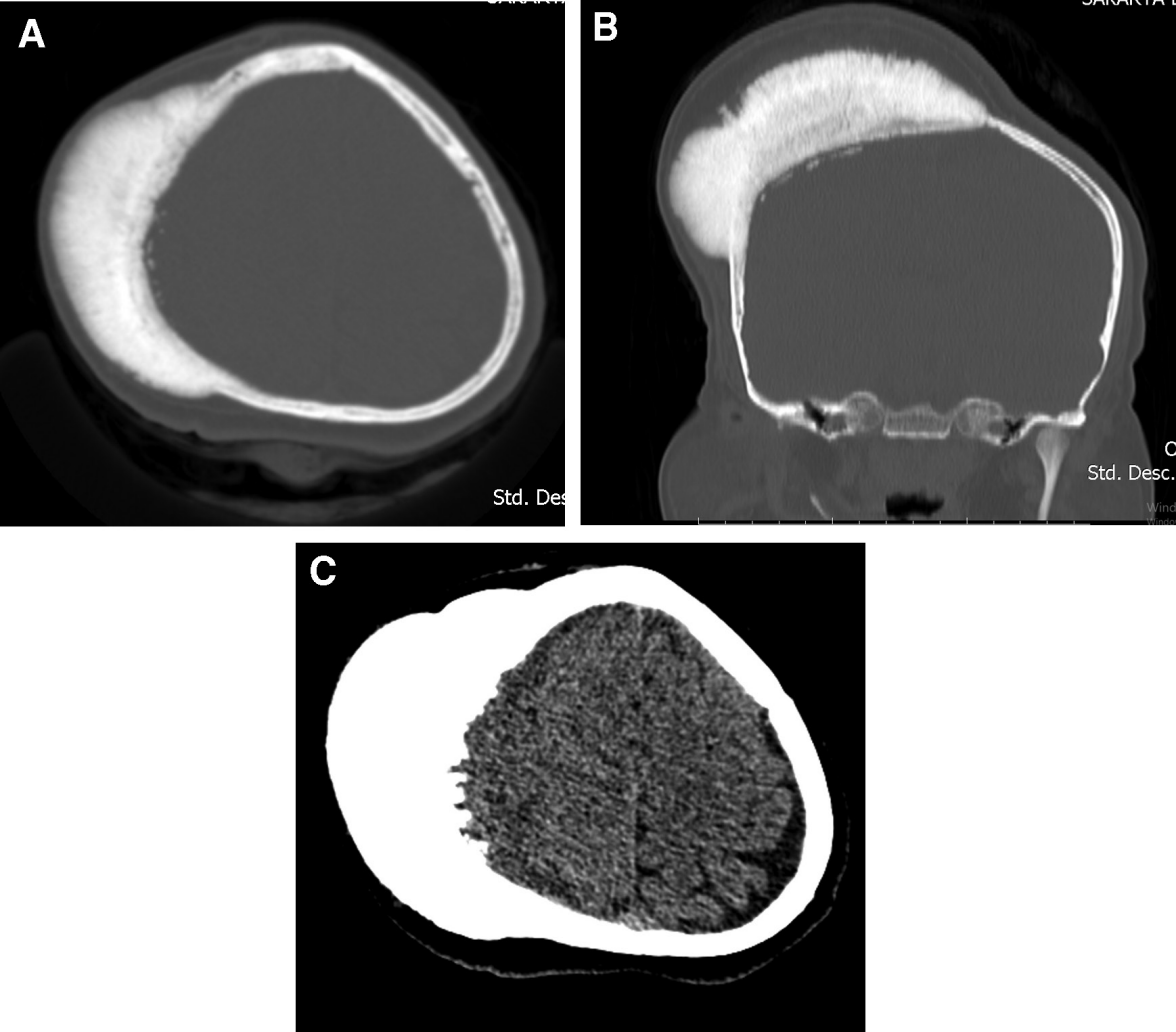
Preoperative cranial computed tomography obtained in 2024. (A) Axial bone-window image showing marked interval enlargement of the right temporoparietal calvarial lesion. (B) Coronal bone-window image demonstrating extensive diploic expansion and cortical thinning. (C) Axial brain-window image showing inner table irregularity abutting the dura without a definite intradural mass component.

Repeat cranial CT in 2024 showed marked interval enlargement of the lesion to 17.6 × 14.3 × 4.4 cm (Fig. 2A–C). The mass involved the diploic space of the right temporoparietal bone and exhibited a heterogeneous mixed osteolytic–sclerotic pattern. The osteolytic component was associated with marked expansion of the calvarium and progressive thinning/destruction of the cortical tables, whereas sclerotic and hyperostotic areas were interspersed within the lesion. The inner table was irregular and focally extended toward the dura, with extradural hyperdense foci suggesting close dural contact; however, no definite intradural mass component was identified on CT. These findings raised the radiologic differential diagnosis of intraosseous meningioma, fibrous dysplasia, and metastatic skull lesion.

### 2.2. Surgical intervention

A right temporoparietal craniectomy was performed. Key intraoperative findings:

- Tumor was poorly marginated and infiltrative within the diploic space.
- Bone resection was carried out up to the dura, where firm adhesions were encountered.
- The dura was not opened, and a thin layer of bone was intentionally preserved over intact dura.
- Total operative time: ~3 hours.
- Estimated blood loss: minimal (no transfusion).

**Figure 3.**
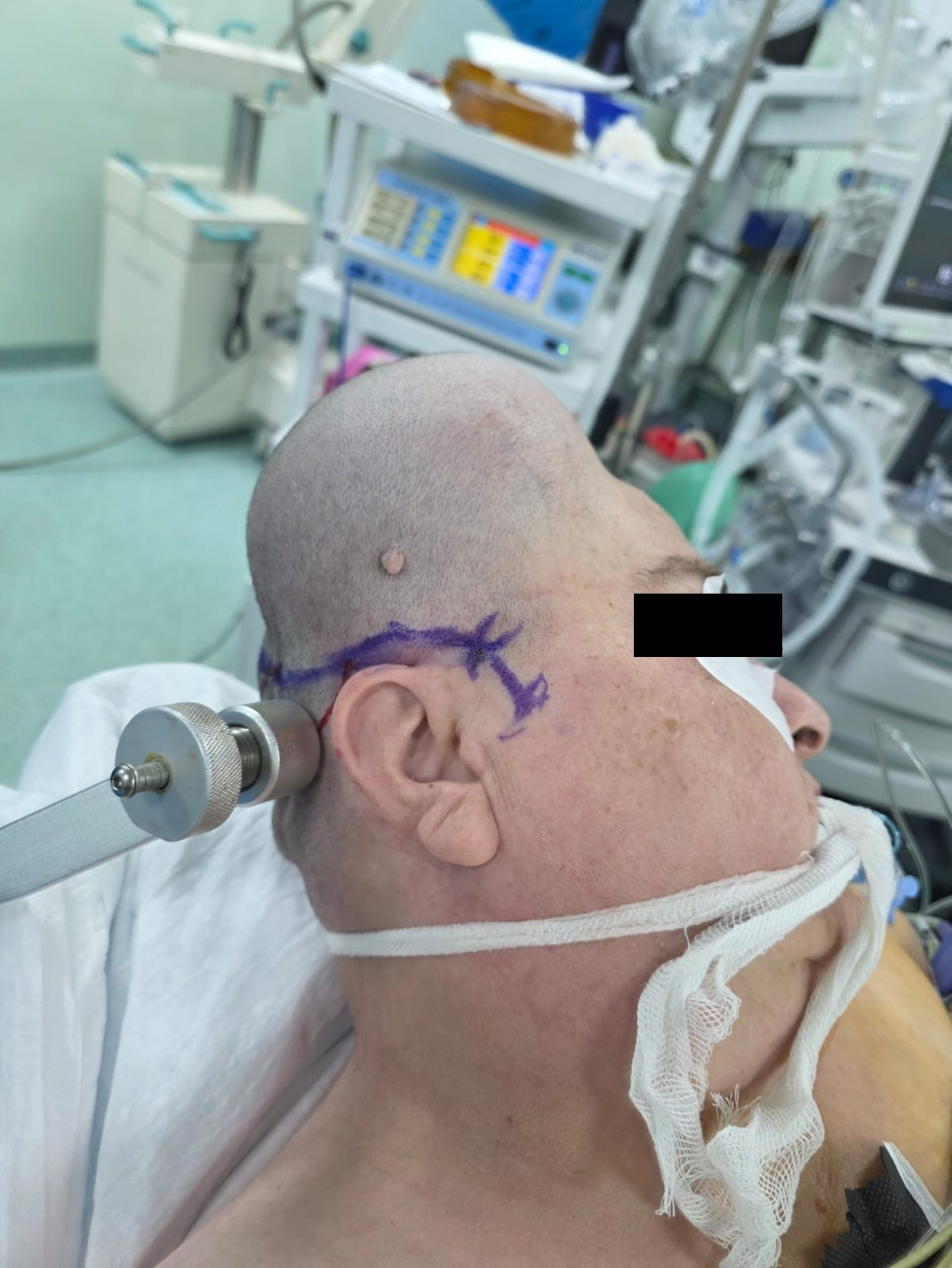
Preoperative clinical photograph showing the patient’s operative position and the external appearance of the large right temporoparietal calvarial mass.

**Figure 4.**
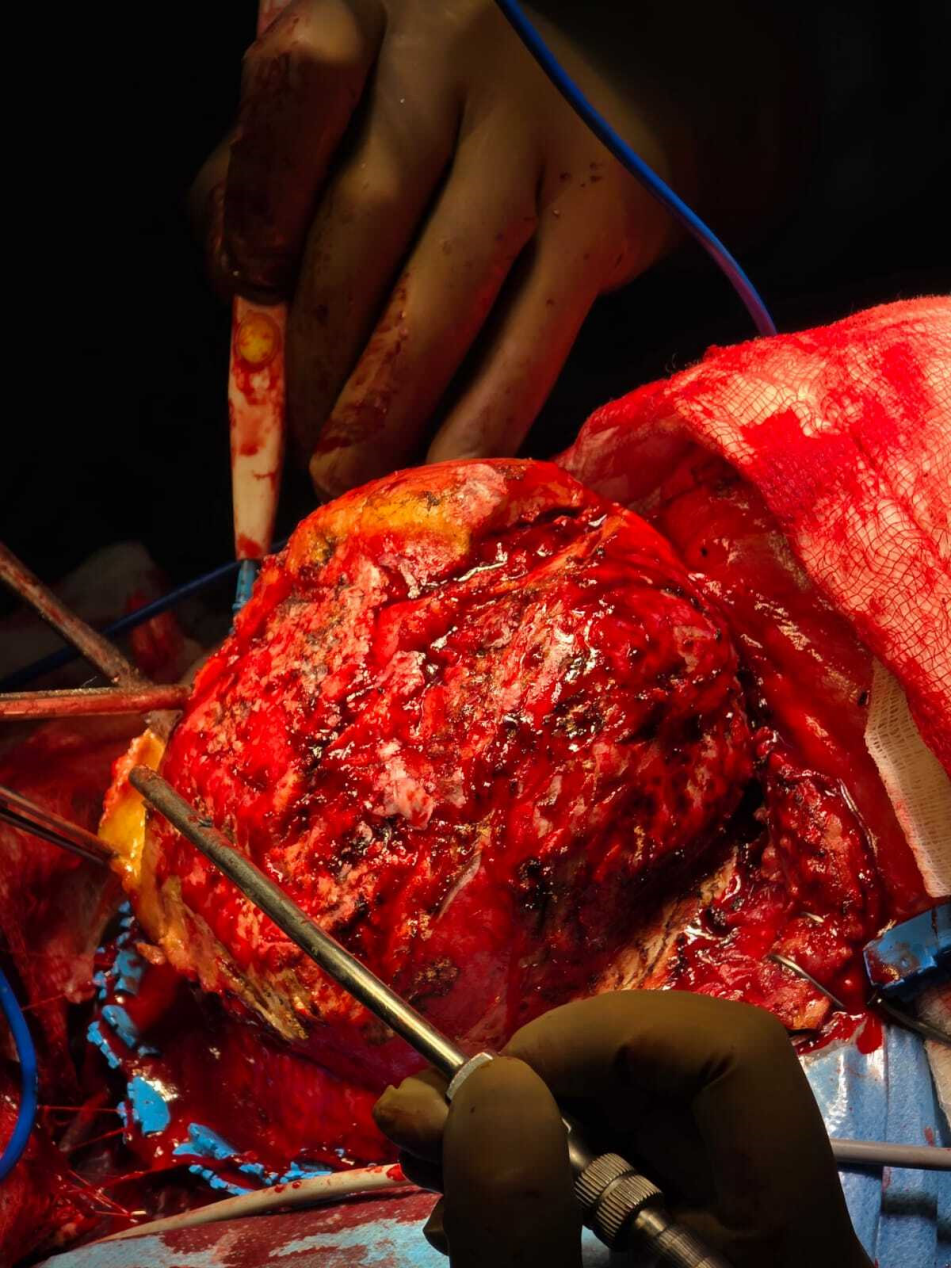
Intraoperative photograph showing the soft tissue overlying the involved calvarial bone after reflection of the skin, subcutaneous tissue, and muscle.

**Figure 5.**
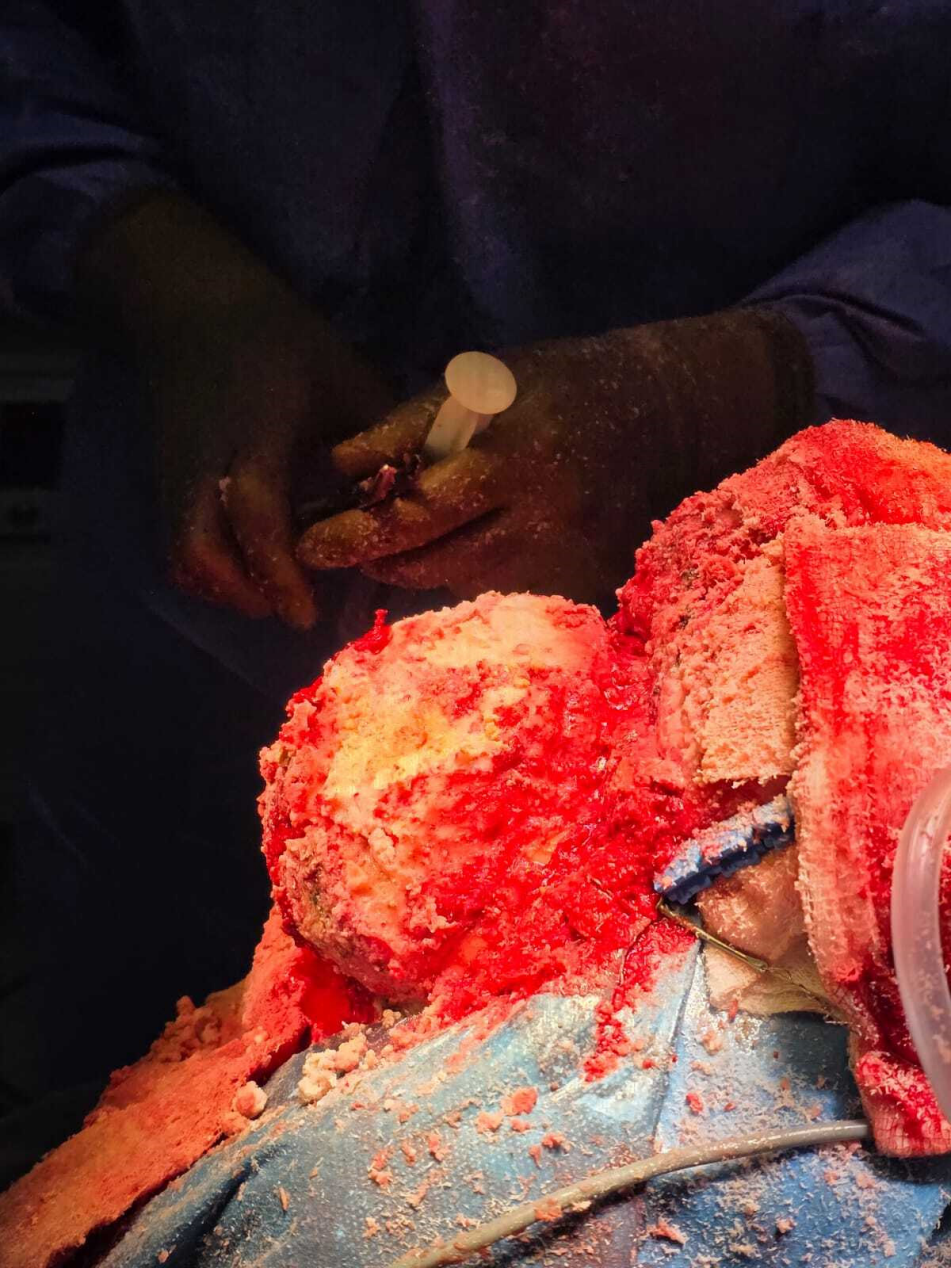
Intraoperative photograph showing the abnormal expanded bone after soft-tissue dissection.

**Figure 6.**
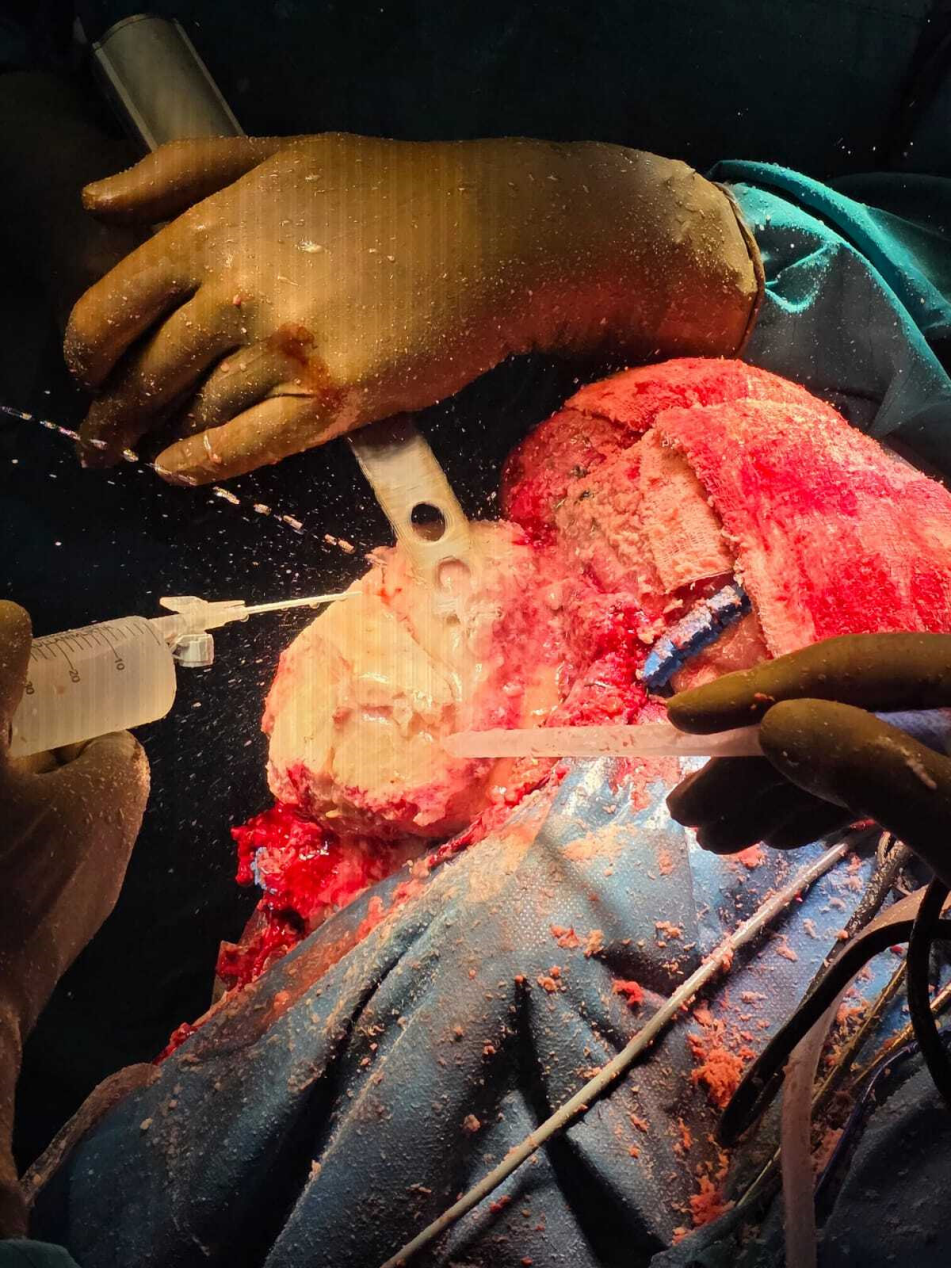
Intraoperative photograph demonstrating the stage of calvarial bone excision.

**Figure 7.**
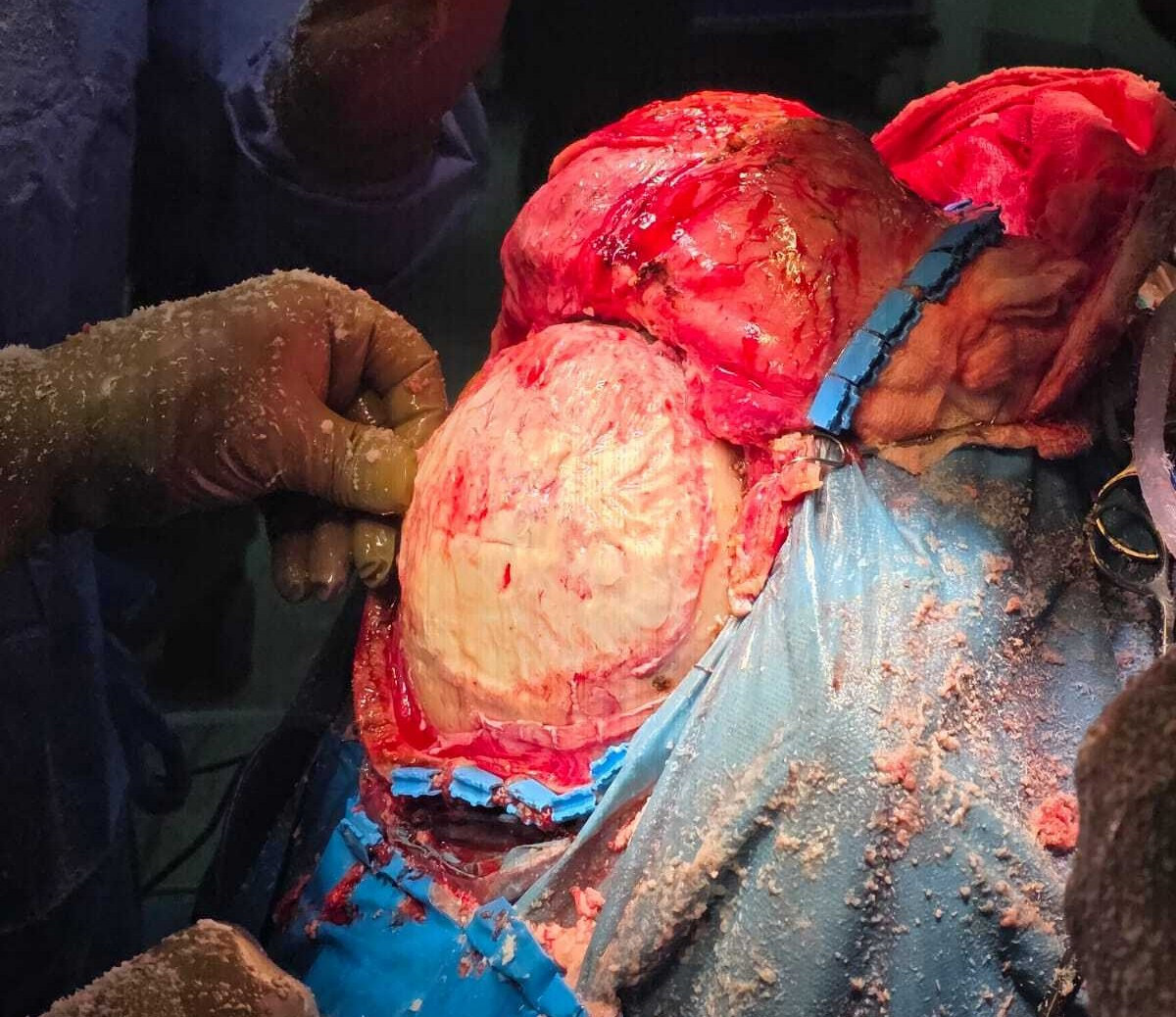
Intraoperative photograph showing the residual thin bony layer adherent to the intact dura after tumor-bearing bone removal.

**Figure 8.**
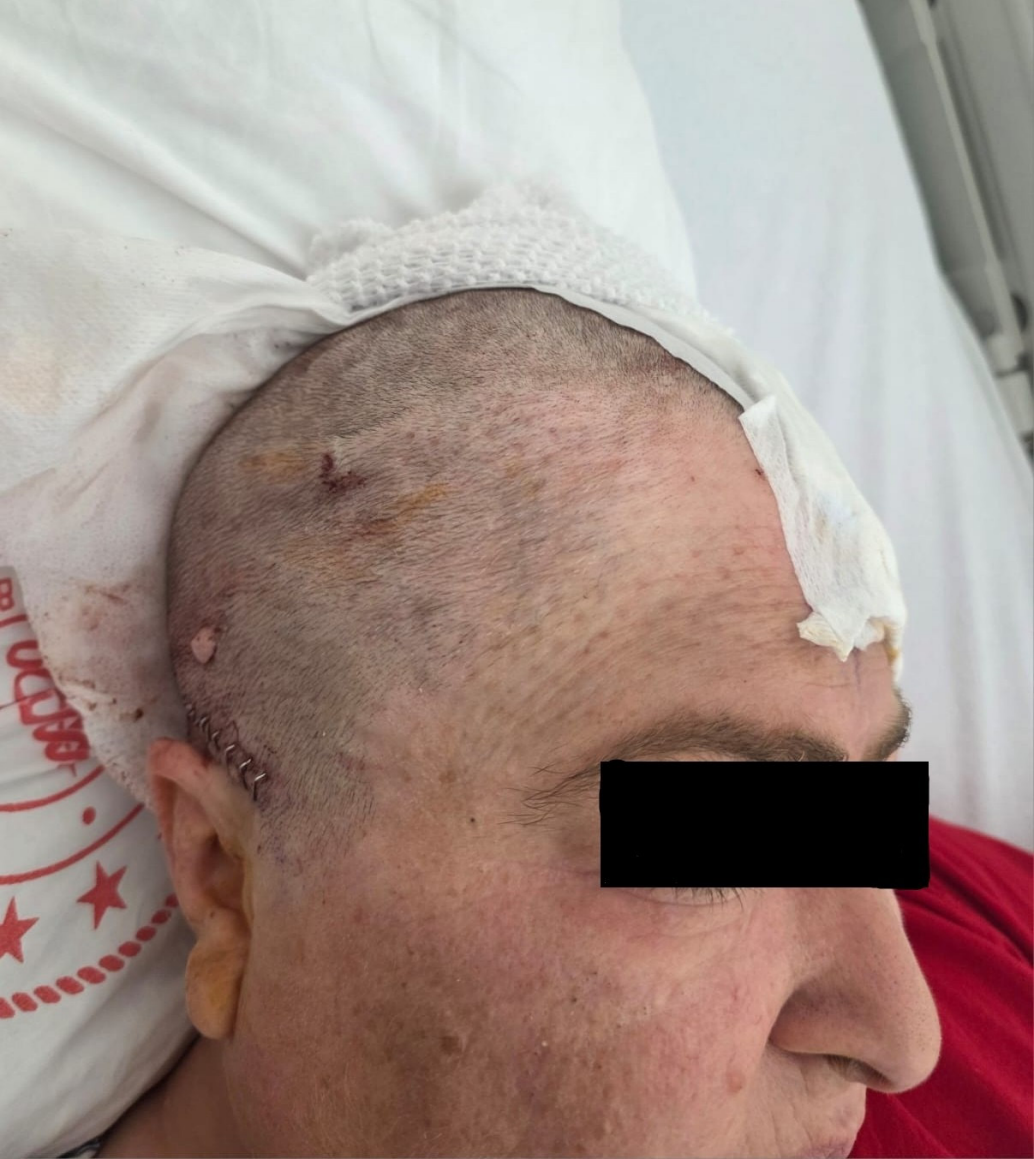
Clinical photograph obtained on postoperative day 1 showing the early postoperative appearance.

The patient’s intraoperative position and the external appearance of the mass, the soft tissue surrounding the bone, the bone tissue, the excision of the bone tissue, the bone tissue adhering to the dura, and the patient on the first postoperative day are shown in Figures 3 to 8.

### 2.3. Histopathology

Microscopy demonstrated meningothelial cells in whorled patterns, without atypia or necrosis. Immunohistochemistry:

- epithelial membrane antigen (EMA)+, Vimentin+, progesterone receptor (PR)+, E-cadherin+
- somatostatin receptor 2 (SST2)−, S100−, pancytokeratin (panCK)−, CD138−
- Ki-67 ≈ 1%

Findings were consistent with ectopic (intraosseous) World Health Organization Grade 1 meningioma.

### 2.4. Postoperative course

The patient recovered uneventfully, with no neurological deficits. Cosmetic contour improved markedly.

One-year follow-up showed no recurrence. Nevertheless, longer follow-up is required to better assess long-term recurrence risk.

**Figure 9.**
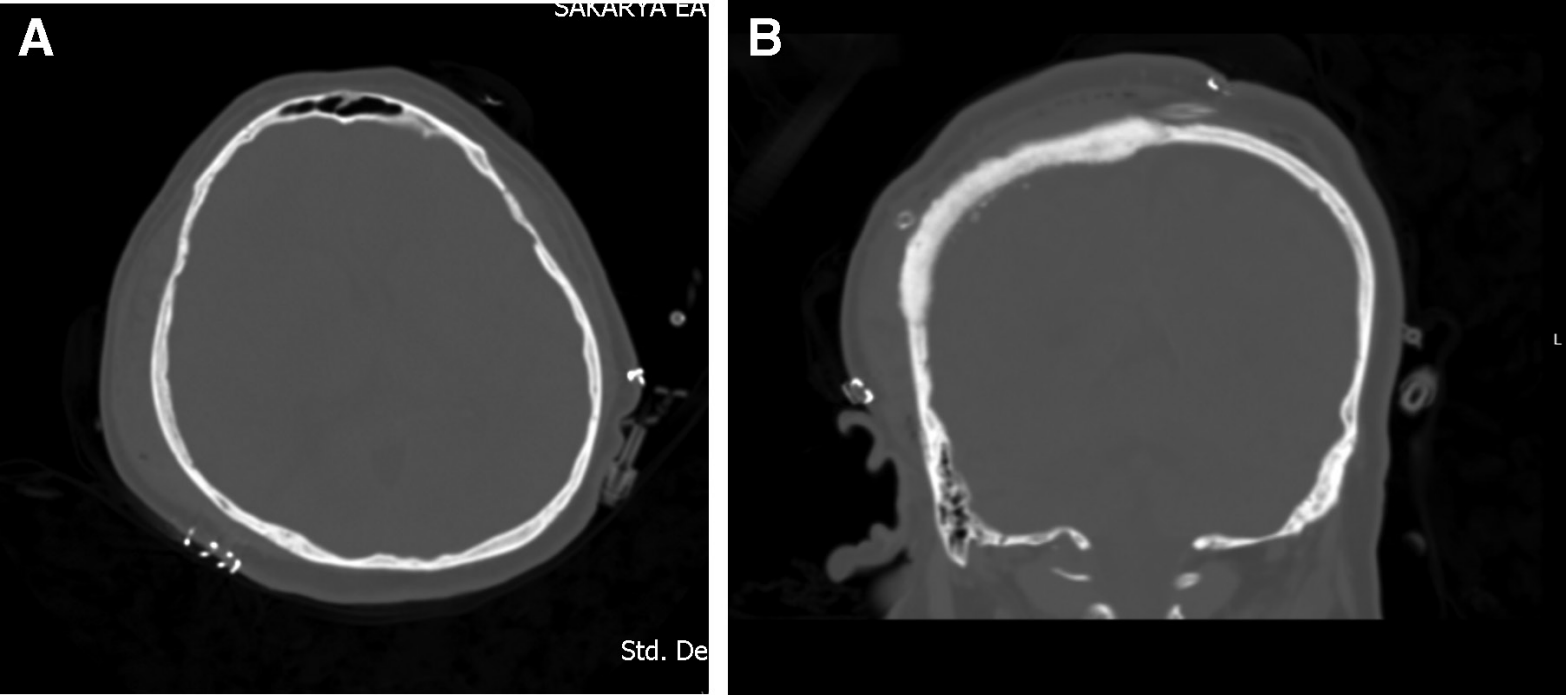
Immediate postoperative cranial computed tomography. (A) Axial bone-window image obtained on postoperative day 1. (B) Coronal bone-window image obtained on postoperative day 1.

**Figure 10.**
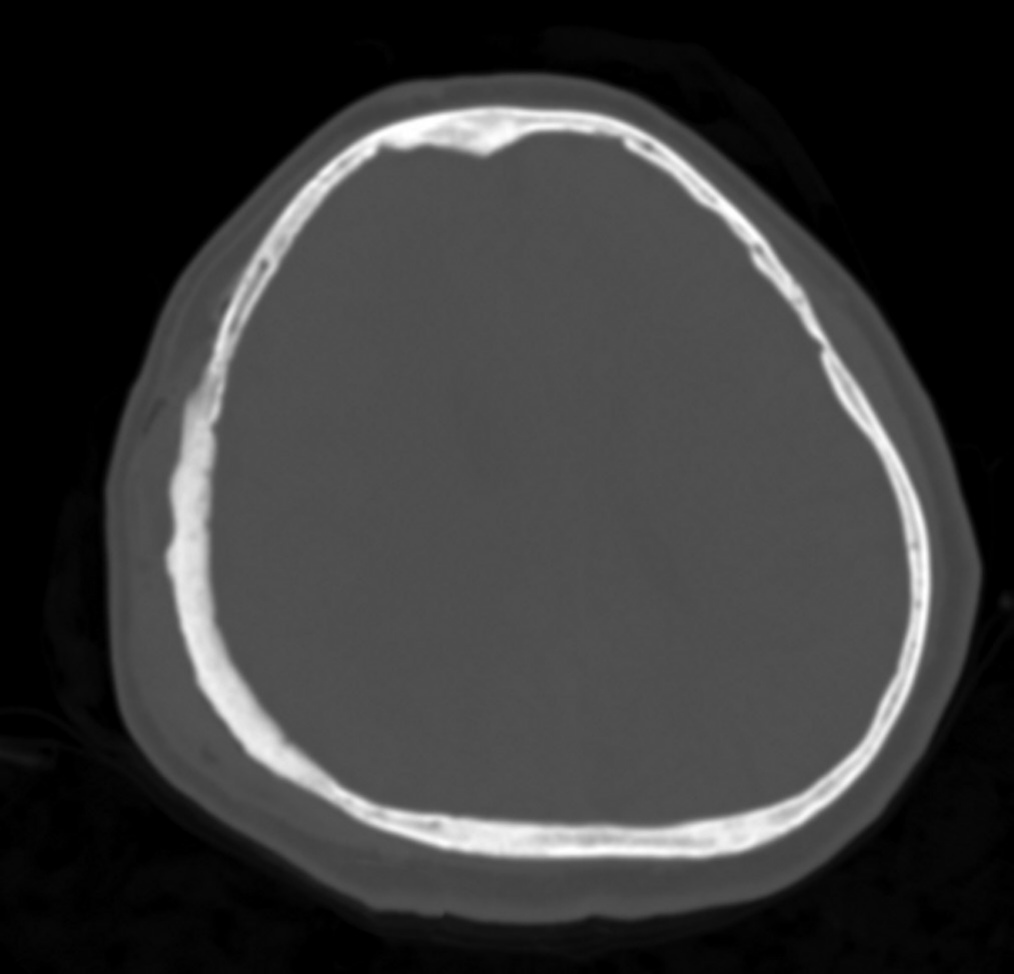
Follow-up cranial computed tomography obtained 1 year after surgery. Axial bone-window image showing no evidence of recurrence or regrowth.

Immediate postoperative and 1-year follow-up CT images are shown in Figures 9A, B and 10.

## 3. Discussion

### 3.1. Epidemiology and pathogenesis

Primary extradural and intraosseous meningiomas are rare, representing <1% of meningiomas.^[1]^ Proposed mechanisms include:

- Arachnoid cell entrapment in cranial sutures
- Embryologic rests of meningothelial tissue
- Mesenchymal metaplasia
- Post-traumatic displacement of arachnoid cells^[6,7]^

The absence of trauma in our patient and the purely diploic origin support a developmental entrapment mechanism.

### 3.2. Radiologic–pathologic correlation

Intraosseous meningiomas often present with hyperostosis, but up to one-third exhibit lytic or mixed patterns.^[1]^ Our patient’s lesion displayed aggressive expansion with cortical destruction – despite benign histology. This dissociation between imaging aggressiveness and biological behavior is well documented.^[8]^

Differential diagnoses include:

- Osteoma
- Fibrous dysplasia
- Eosinophilic granuloma
- Metastases (breast, prostate, thyroid)
- Hemangioma
- Osteosarcoma

CT characteristics such as irregular infiltration, diploic expansion, and dural-facing erosions favor intraosseous meningioma over benign bone lesions.^[9]^

### 3.3. Growth dynamics

The lesion expanded from **~**6 cm to ~18 cm over 5 years – one of the fastest documented growth trajectories in benign intraosseous tumors. Although World Health Organization Grade 1 lesions typically grow slowly, intraosseous forms may show accelerated local osteolytic activity unrelated to proliferative indices.^[10]^

Low Ki-67 (≈1%) in our case suggests benign histology, yet the rapid cranial expansion indicate**s** mechanical and osteolytic remodeling rather than cellular proliferation.^[11]^

### 3.4. Histological and molecular considerations

Benign meningiomas express EMA, Vimentin, and PR. Loss of PR or high Ki-67 suggests aggressive variants.^[12]^

This lesion’s EMA+/PR+/Ki-67 low profile aligns with Grade 1 biology.

Although molecular testing was not performed, literature shows some intraosseous meningiomas harbor SMO, AKT1, or NF2 mutations, influencing behavior and recurrence risk.^[13,14]^ Future studies may clarify genomic roles in ectopic meningiomas.

### 3.5. Surgical strategy

Radical excision of involved bone is the recommended treatment.^[15]^ When dural invasion is suspected, dural resection may be necessary; however, firm dural adhesion does not necessarily indicate true dural invasion, and dural preservation may be appropriate when direct invasion is not evident.^[1,16]^

In our case, the dura was preserved safely with no recurrence at 1 year – consistent with outcomes in tumors with low Ki-67 and intact dura.

Cranioplasty is optional depending on defect size. The patient maintained adequate contour after resection.

### 3.6. Prognosis and follow-up

Recurrence rates for intraosseous meningiomas range from 0% to 22% and are influenced by the extent of resection, dural involvement, histological grade, and proliferative activity.^[8,17]^

In our patient, complete resection of the involved calvarial bone, preservation of an apparently uninvolved dura, and a low Ki-67 labeling index suggest a favorable biological profile. However, the relatively short follow-up period is an important limitation of this report. Although no recurrence was detected at 1 year, this interval is insufficient to determine durable long-term tumor control in such a large lesion. In addition, the absence of preoperative magnetic resonance imaging limited more precise preoperative assessment of dural and possible adjacent soft-tissue involvement. Accordingly, prolonged clinical and radiological surveillance is warranted before definitive conclusions regarding long-term prognosis can be made.

## 4. Conclusion

Giant intraosseous meningiomas, although extremely rare, should be considered in the differential diagnosis of large calvarial masses. Their radiologic appearance may mimic primary bone neoplasms or metastases, emphasizing the importance of comprehensive evaluation and serial imaging.

This case demonstrates that even massive, rapidly enlarging intraosseous tumors can be managed effectively through carefully planned bone resection while preserving the dura when invasion is not evident. The benign histopathological profile and absence of recurrence at 1 year are encouraging; however, longer follow-up is necessary before durable long-term control can be confirmed.

Early recognition, accurate radiologic interpretation, and individualized surgical strategies are essential to optimize aesthetic and functional outcomes in this uncommon meningioma variant.

## Author contributions

**Conceptualization:** Muhammed Ömer Bakaç.

**Data curation:** Muhammed Ömer Bakaç, Şengül Koçer.

**Formal analysis:** Muhammed Ömer Bakaç.

**Investigation:** Muhammed Ömer Bakaç, Şengül Koçer.

**Methodology:** Mustafa Kaya, Muhammed Ömer Bakaç.

**Project administration:** Mustafa Kaya, Muhammed Ömer Bakaç.

**Resources:** Muhammed Ömer Bakaç, Şengül Koçer.

**Software:** Muhammed Ömer Bakaç, Şengül Koçer.

**Supervision:** Mustafa Kaya.

**Validation:** Mustafa Kaya, Muhammed Ömer Bakaç.

**Visualization:** Muhammed Ömer Bakaç.

**Writing – original draft:** Muhammed Ömer Bakaç.

**Writing – review & editing:** Mustafa Kaya, Muhammed Ömer Bakaç.
